# PI3K/Akt/Nrf2 mediated cellular signaling and virus-host interactions: latest updates on the potential therapeutic management of SARS-CoV-2 infection

**DOI:** 10.3389/fmolb.2023.1158133

**Published:** 2023-06-01

**Authors:** V. S. Lekshmi, Kumari Asha, Melvin Sanicas, Abhila Asi, U. M. Arya, Binod Kumar

**Affiliations:** ^1^ Department of Antiviral Research, Institute of Advanced Virology, Thiruvananthapuram, Kerala, India; ^2^ Department of Microbiology and Immunology, Chicago Medical School, Rosalind Franklin University of Medicine and Science, North Chicago, IL, United States; ^3^ Clover Biopharmaceuticals, Boston, MA, United States

**Keywords:** Coronavirus, SARS-CoV-2, pandemic, COVID-19, Nrf2, oxidative stress, inflammation, PI3K/Akt pathway

## Abstract

The emergence and re-emergence of viral diseases, which cause significant global mortality and morbidity, are the major concerns of this decade. Of these, current research is focused majorly on the etiological agent of the COVID-19 pandemic, SARS-CoV-2. Understanding the host response and metabolic changes during viral infection may provide better therapeutic targets for the proper management of pathophysiological conditions associated with SARS-CoV-2 infection. We have achieved control over most emerging viral diseases; however, a lack of understanding of the underlying molecular events prevents us from exploring novel therapeutic targets, leaving us forced to witness re-emerging viral infections. SARS-CoV-2 infection is usually accompanied by oxidative stress, which leads to an overactive immune response, the release of inflammatory cytokines, increasing lipid production, and also alterations in the endothelial and mitochondrial functions. PI3K/Akt signaling pathway confers protection against oxidative injury by various cell survival mechanisms including Nrf2-ARE mediated antioxidant transcriptional response. SARS-CoV-2 is also reported to hijack this pathway for its survival within host and few studies have suggested the role of antioxidants in modulating the Nrf2 pathway to manage disease severity. This review highlights the interrelated pathophysiological conditions associated with SARS-CoV-2 infection and the host survival mechanisms mediated by PI3K/Akt/Nrf2 signaling pathways that can help ameliorate the severity of the disease and provide effective antiviral targets against SARS-CoV-2.

## Introduction

Coronaviruses (CoVs) are enveloped, non-segmented positive sense, single stranded RNA viruses belonging to the family Coronaviridae. They are amongst the largest group of viruses categorized into four genera: Alphacoronaviruses, Betacoronaviruses, Gammacoronaviruses, and Deltacoronaviruses, infecting different types of animals that can cause mild to severe respiratory and gastrointestinal complications in humans (Capron, 1987). Historically these viruses were linked to the human CoVs (229E and OC43) that caused mild upper respiratory tract diseases. The emergence and re-emergence of Coronavirus diseases in the twenty-first century has sparked public concern since 2002 with the severe acute respiratory syndrome Coronavirus (SARS-CoV) (Ksiazek et al., 2003) and the Middle East respiratory syndrome Coronavirus (MERS-CoV) outbreaks (Zaki et al., 2012; Cui et al., 2019). Severe acute respiratory syndrome coronavirus 2 (SARS-CoV-2) is a highly transmissible novel virus belonging to the family Coronaviridae (betacoronavirus 2B lineage), together with SARS-CoV and MERS-CoV viruses that caused previous outbreaks. SARS-CoV-2 is the etiological agent of the coronavirus disease (COVID-19) epidemic that emerged in late 2019 in Wuhan, China. On 11 March 2020, the World Health Organization (WHO) declared COVID-19 a pandemic with a high transmission rate, mortality, and morbidity (Team, 2020). Although a great deal of research has been conducted regarding viral emergence and transmission, there is very little available information explaining the pathophysiology and viable therapeutic options. In majority of cases, the infected patients do not require any special medical intervention, however in about 20% of the COVID-19 cases, patients do necessitate hospitalizations (Wu and McGoogan, 2020). Most of the hospitalizations are accounted to hyperinflammation damaging organs and endothelium of blood vessels, thrombosis and immunosuppression with a possible role in latent long COVID or post-COVID conditions (Ackermann et al., 2021; Zhu et al., 2022). Several reports in the past have already documented that oxidative stress and inflammation mutually reinforce each other and the same have also been observed in COVID-19 patients (Jensen et al., 2021). Although elevated levels of reactive oxygen species (ROS) have detrimental consequences on cell viability and many viruses have still evolved to induce oxidative stress for their own benefit of replication inside cells (Lee, 2018). Another important event associated with viral infection is the host lipid metabolism that plays vital role in oxidative stress, inflammatory response and thrombotic complications associated with SARS-CoV-2 infection (Casari et al., 2021). Lipids also form the structural foundations of viral and cellular membranes and thus during viral infection, viruses hijack host cellular signaling and lipid biogenesis to produce lipids and other metabolites in favour of the virus life cycle. Lipidomic approaches may provide valuable insights into the host response to COVID-19 and studies highlighting such roles may provide potential therapeutic targets (Abu-Farha et al., 2020). The virus-host harmonious balance is the key to the survival of these viruses. Since many of the antiviral signaling pathways are initiated due to infection induced oxidative stress, it is imperative to understand the mechanistic details of how host cells maintain the redox balance. A clear understanding will allow to effectively modulate the antiviral targets.

To date, there is no specific treatment strategy for curing COVID-19, however, new research and developments have paved pathways to select potent antiviral agents that can be used effectively as therapeutics against COVID-19. From the previous findings, it can be deduced that one of the best and most efficient cellular targets for the management of SARS-CoV-2 infection and pathogenicity could be the PI3K/Akt/Nrf2 signaling pathways. PI3K (phosphatidylinositol 3-kinase) is a family of enzymes that are involved in cell survival and intracellular trafficking. Nrf2 (nuclear factor erythroid 2–related factor 2) is a key transcription factor that acts as a sensor of oxidative stress and an important regulator of antioxidant defense mechanism via modulating the transcription of more than 200 cytoprotective genes (Tebay et al., 2015). Previous studies have shown that multiple viruses including Herpes simplex virus, Porcine circovirus, Influenza A viruses, vaccinia and cowpox viruses utilize the PI3K/Akt signalling pathways for replication and establising successful infection (Ehrhardt et al., 2007; Soares et al., 2009; Wei et al., 2012; Eaton et al., 2014). Studies have also reported that the PI3K pathway is activily involved in the endocytic uptake of influenza viruses (Ayllon et al., 2012) and ebola viruses (Saeed et al., 2008) thus demonstrating the extent of involvement of signalling pathways in viral infections. PI3K/Akt pathway has been shown to play a critical role in regulating SARS-CoV-2 entry (Shou et al., 2020) and evidence from other studies further suggest that the PI3K/Akt signaling pathway also inhibits NF-κB and subsequently reduces the expression of inflammatory cytokines (Li et al., 2021). Nrf2 and NF-κB has also been known to transcriptionally co-regulate the response of cells to oxidative stress and inflammation via the PI3K/Akt/Nrf2 signaling pathway (Lekshmi et al., 2019), thus making it a crucial target to develop host directed antiviral strategies against SARS-CoV2 and other related viruses.

A comprehensive analysis of all data suggests that PI3K/Akt/Nrf2 signaling could be a powerful tool to manage SARS-CoV-2 infection via antioxidant, anti-inflammatory, and lipid metabolism regulation. In this review, possible cellular targets and molecular mechanisms involved in SARS-CoV-2 infection, as well as therapeutic approaches to treat its pathophysiological complications, are discussed with special emphasis on PI3K/Akt/Nrf2 pathway.

### Viruses hijack PI3K/Akt/Nrf2 pathway for survival

Several viruses exploit the host metabolic pathways in order to meet their needs of survival. The phosphatidylinositol 3-kinases (PI3K)-Akt pathway is one such signaling event that is central to metabolism and other cellular functions as well as a common target of many viruses (Cooray, 2004; Buchkovich et al., 2008; Diehl and Schaal, 2013). Although the PI3Ks belong to a large family of lipid kinases belonging to 3 classes: class 1 (1A and 1B), class II, and class III; the PI3K-Akt pathway falls within the class 1A PI3Ks. The class 1A PI3Ks get activated directly or indirectly via small GTPase RAS. The PI3K activation further phosphorylates and activates its most prominent effector Akt which then localizes to the plasma membrane. Viruses have evolved to utilize this pathway for successful entry into target cells or trafficking through the cytoplasm (Saeed et al., 2008; Feng et al., 2011; Fujioka et al., 2011; Izmailyan et al., 2012).

Nrf2, nuclear factor (erythroid-derived 2) -like 2, is a cytoprotective transcription factor belonging to the cap´n´collar basic leucine zipper family that binds to antioxidant response element (ARE) for regulating the transcription of genes encoding proteins which maintain cellular redox homeostasis and metabolic balance (Cuadrado et al., 2019). Under normal conditions, Nrf2 is maintained in an inactive state in the cytosol by binding with KEAP 1 (Kelch-like ECH-associated protein 1), an adaptor subunit of Cullin 3-based E3 ubiquitin ligase which acts as a sensor of oxidative stress. ROS, by modifying the specific cysteine residues, inactivates KEAP1 and thereby releases Nrf2 into the nucleus to induce transcription of Nrf2-responsive genes by binding with ARE. This in turn activates ARE-dependent gene expression of a series of cytoprotective and antioxidative proteins including heme oxygenase-1 (HO-1), glutathione peroxidase 1, glutathionine S-transferase (GST), glutathione reductase (GR), and superoxide dismutase (SOD), catalase (CAT), NAD(P) H dehydrogenase, quinone 1 (NQO1) and c-glutamylcysteine synthetase (Sun et al., 2021a).

The pathophysiology of respiratory viral infections generally involves a redox imbalance or oxidative stress that is associated with the release of cytokines, inflammation and cell death. Studies have shown crucial roles of overproduction of ROS in virus replication and virus-associated diseases (Gu and Korteweg, 2007). Since excessive oxidative stress can be detrimental to the host cells, several viruses maintain an optimal level of oxidative stress, enough to support its replication without killing cells, by manipulating the Nrf2 pathway. When ROS are released upon viral infection, the host cells activate an antioxidative defense mechanism, in which the Nrf2 pathway acts as a first line of defense for cytoprotection and detoxification. Virus-induced modulation of the host antioxidative response have been reported to be an important factor in the progression of several viral diseases. Recently several studies have also reported that groups of clinically relevant viruses can regulate the Nrf2 pathway in both positive and negative manner (Lee, 2018).

Human immunodeficiency virus type 1 (HIV-1), the etiological agent of acquired immunodeficiency syndrome (AIDS) is also linked with the development of neurocognitive disorders. The viral protein, gp120 is known for its causative role in the HIV-1-associated neurodegeneration through induction of oxidative stress. Based on studies conducted in HIV infected astrocytes, the use of Nrf2 activators was suggested as a promising approach to enhance lung innate immunity in HIV patients (Reddy et al., 2012). In 2012, Zhang et al., demonstrated that a major catechin from tea, Epigallocatechin-3-O-gallate (EGCG), was able to improve the cellular alterations induced by oxidative stress associated with Tat-induced HIV-1 transactivation by regulating nuclear levels of Nrf2 and NF-κB. The findings make the Nrf2 pathway the prime therapeautic target (Zhang et al., 2012). Hepatitis C Virus (HCV), responsible for chronic hepatitis, exerts differential effects on the Nrf2 pathway depending on the cellular context and level of oxidative stress. Numerous HCV proteins, including the core, NS3, and NS5A, cause hepatocellular damage as a result of oxidative stress. The production of ROS during HCV infection promotes the phosphorylation and nuclear translocation of Nrf2, which activates target genes such as HO-1 and glutamylcysteine synthetase heavy subunit (γGCSH) (Carvajal-Yepes et al., 2011; Ivanov et al., 2011). Numerous cellular kinases have been implicated in the phosphorylation and activation of Nrf2, including PI3K-Akt, JNK, ERK1/2, p38 MAPKs, and protein kinase C (PKC). In light of these findings, activation of Nrf2 pathway was suggested as one possible mechanism for HCV-infected cells to survive (Carvajal-Yepes et al., 2011; Ivanov et al., 2011).

Similarly, influenza viruses induce infection mainly through oxidative stress and respiratory inflammation. In addition, influenza viruses have also been shown to stimulate apoptosis and cytotoxicity in alveolar epithelial cells, as demonstrated by an increase in caspase 1, caspase 3, and the proinflammatory cytokine IL-8 via activation of Nrf2 pathway by facilitating nuclear translocation of Nrf2 and subsequent expression of Nrf2-target genes such as HO-1 (Kosmider et al., 2012). The suppression of Nrf2 gene was also found to enhance the replication of influenza virus which was reversed by the pharmacological induction of Nrf2 via EGCG supplementation (Kesic et al., 2011). In a proteomic analysis performed by Simon and colleagues, Nrf2 was found to be negatively affected by influenza virus infection. Thus, like HCV infection, influenza virus infection has also been found to induce differential antioxidative responses depending on cellular context (Simon et al., 2015). Likewise, positive or negative regulation of Nrf2 via PI3K/Akt or other signaling pathways through pharmacological modulators have shown to regulate the infection of various viruses including RSV, HCV, HBV, Herpes, DENV and Zika virus (Cho et al., 2009; Zhu et al., 2010; Schachtele et al., 2012; Huang et al., 2017).

The respiratory syndrome caused by SARS-CoV-2 continues to be a major healthcare concern around the globe because of no specific treatment availability for COVID-19. Since the treatments for COVID-19 are known to suppress the symptoms, modulating signaling pathways via therapeutic targets could be important for managing the disease severity. The PI3K/Akt signaling pathway has been identified as a novel therapeutic target against SARS-COV-2 infection due to its involvement in virus entry and host immune response. ACE2 and CD147 are known to be the prime entry receptors for SARS-COV-2 (Hoffmann et al., 2020; Wang et al., 2020). The reduced cell surface expression of ACE2 during infection results in angiotensin 2 accumulation, which upon binding to AT1R (angiotensin 2 receptor type 1) activates the inflammatory pathway via NF-κB signaling. Recent studies have demonstrated that CD147 and furin, as well as clatherin-mediated endocytosis, also induce P13K/Akt signaling (Khezri, 2021). A recent study has also shown that SARS-CoV-2 S protein can modulate inflammatory responses via the PI3K/Akt pathway to allow propagation of virus at early stages of infection (Al-Qahtani et al., 2022).

Apart from being exploited by the viruses during their life cycle, the PI3K/Akt pathway also serves to counteract viral invasion by inducing phosphorylation of IFN regulatory factor 3 (IRF3) and type I interferons (IFN-I) (Schabbauer et al., 2008; Joung et al., 2011). Infection with many double/single stranded viruses also activate the PI3K/Akt for TLR-mediated tyrosine phosphorylation and RIG-I dependent activation of the IRF3 (Sarkar et al., 2004; Yeon et al., 2015). A better understanding of how virus-induced lipid kinase pathways and oxidative stress communicates with the host’s antioxidative response, will provide insights into potential antiviral therapeutics that can be discovered and developed for efficient viral disease management.

### Structure of SARS-CoV-2, its life cycle and host cell invasion

**FIGURE 1:**
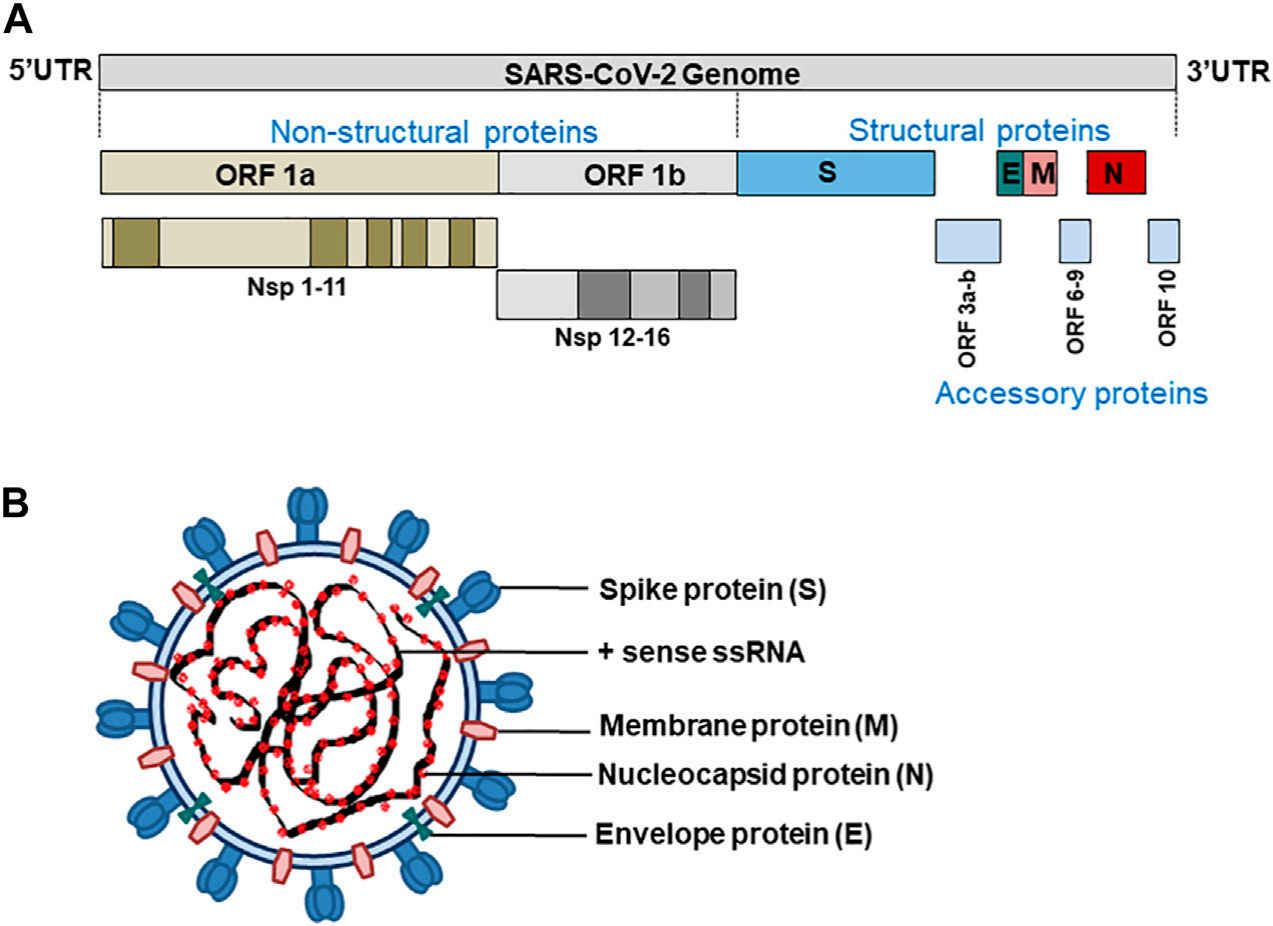
**(A)** SARS-CoV-2 genome **(B)** structure of SARS-CoV-2.

Coronaviruses have a genome size of 27–32 kb, which is generally larger than any other RNA viruses. SARS-CoV-2 has a genome size of approximately 29.9 kb and codes for 4 structural proteins S (spike glycoprotein), N (nucleocapsid protein), M (membrane protein), and E (Envelope protein), 16 non-structural (Nsp1-16), and nine accessory proteins (Orf3a, Orf3b, Orf6, Orf7a, Orf7b, Orf8, Orf9b, Orf9c, Orf10) (Gordon et al., 2020; Lu et al., 2020; Mariano et al., 2020) (Figure 1).

The proteins S, M, and E make up the viral envelope. The invasion of a host cell, the first step in SARS-CoV-2 infection, is mediated by the transmembrane spike protein (180–200 kDa). This allows SARS-CoV-2 virions to attach to the host cell membrane receptors (ACE2) and invade those cells by subsequent fusion of viral and host cell membranes or endocytosis.

The nucleocapsid protein N is recruited at the replication-transcription complex by Nsp3 where it plays a multifaceted role in the infection cycle of SARS-CoV-2. The N protein bind to and package the viral RNA to form ribonucleoprotein (RNP) complexes that locates in the internal face of the viral membrane as a separate layer from the envelope proteins S, M and E (Chang et al., 2014). N protein has two structured domains that allow it to carry out many functions during the viral life cycle, such as virion assembly, RNA replication/transcription, and immune system interference. Since the domains of the N protein are separated by a long flexible linker, it has a high degree of conformational freedom (McBride et al., 2014).

Similarly, the primary function of the membrane protein M, which is embedded by three transmembrane helices, is to drive the assembly of virions to the host cell and maintain other structural proteins at the budding site, and recruit the same by promoting membrane curvature (Neuman et al., 2011). Also, SARS-CoV-2-M proteins are reported to have high pro-apoptotic properties and induce apoptosis by disrupting the interaction of PDK1 (3-phosphoinositide-dependent protein kinase 1) with cell-survival protein PKB (protein kinase B)/Akt in cells expressing M-protein (Tsoi et al., 2014). Like M protein, SARS-CoV-2 envelope protein E also shows oligomerization properties. Coronavirus E protein has only one transmembrane domain that can self-interact to form ion channels and can also establish interactions with the nucleocapsid protein N (Stertz et al., 2007). The M protein oligomerizes at the membrane of the intermediary compartment of the endoplasmic reticulum and Golgi. The interaction of the C-terminus of E with M guides the recruitment of E and initiate virus budding into the host cells (Pervushin et al., 2009; Schoeman and Fielding, 2019).

In response to the viral infection, a “cytokine storm” (also known as hypercytokinemia) is triggered to induce further inflammatory changes in the pneumocytes. Excessive inflammation and apoptosis ultimately cause lung damage. The released viruses after cell apoptosis, further infects the adjacent type 2 alveolar epithelial cells in the same manner, resulting in acute respiratory distress syndrome (Jackson et al., 2022). Even though respiratory epithelial cells are the prime target for SARS-CoV-2, both direct and indirect cellular alterations due to virus replication, host response, and the triggered inflammatory and hypercoagulative consequences make the condition more lethal (Bezerra et al., 2022). COVID-19 patients mainly exhibit the viral nucleocapsid and spike proteins as main immunogens, and plasma or serum quantitative measurements in SARS-CoV-2 patients showed the N protein to be more sensitive to the adaptive immune response than the spike protein. This makes it an excellent indicator of early disease development (Burbelo et al., 2020).

### SARS-CoV-2 infection and oxidative stress

Elevated ROS production and higher concentrations of oxidized biomolecules have been reported in the alveolar epithelium and endothelium of patients infected with viruses such as influenza (Buffinton et al., 1992), rhinovirus (Martinez et al., 2016), respiratory syncytial virus (RSV) (Biagioli et al., 1999), and many other viruses, however different viruses are known to employ diverse molecular mechanisms to exhibit these cellular effects. While maintaining a proper redox homeostasis is very important for the regulated balance of viral-induced ROS-activated immune cell signal transduction, its excessive production may further induce an impaired immune response, inflammatory reactions, mitochondrial dysfunction, and apoptosis impacting the disease pathogenesis (Chernyak et al., 2020). Mitochondria is the major producer of ROS (mtROS) in non-immune cells like endothelial cells while NADPH oxidase (NOX) and xanthine oxidase are the major sources of ROS in immune cells (Vorobjeva et al., 2017). Most viruses induce oxidative stress in order to facilitate viral replication in the host cell by activating innate immunity via NF-κB-dependent cytokine production. RSV is also reported to induce ROS production and cytokine burst in host cells. To control the ROS levels these viruses are known to acquire the ability to manipulate Nrf2 dependent antioxidant pathway in their favor. RSV ameliorates glutathione (GSH) levels and increases lipid peroxidation in type II epithelial cells of the airway and human alveoli resulting in the downregulation of the Nrf2 pathway. This in turn reduces the expression of Nrf2-dependent target genes; superoxide dismutase (SOD), catalase (CAT), hemoxigenase 1 (HO-1/HMOX1), glutathione S-transferase (GST), and glutathione peroxidase (GPx), and triggers interferon (IFN) and Toll-like receptor (TLR) pathway to combat the virus infection (Jamaluddin et al., 2009). The mechanism however, is different for the influenza virus. The influenza virus induces oxidative stress but it also favors translocation of Nrf2 and thereby activates the antioxidant defense mechanism for its survival in the host cells (Imai et al., 2008).

Oxidative stress is reported as the agent provocateur behind most viral infections and thus the host cell signaling activation accompanied by oxidative stress may have a profound impact on the pathogenesis of COVID-19 and related disorders. A recent study found that the activation of Nrf2/HMOX1 significantly suppressed SARS-CoV-2 replication through production of the metabolite biliverdin in different cell types. The same study also demonstrated that the virus impaired the Nrf2/HMOX1 axis through its NSP14 which interacted with the catalytic domain of the NAD-dependent deacetylase Sirtuin 1 (SIRT1) thereby inhibiting the Nfr2/HMOX1 pathway. While this finding revealed the crucial role of a viral protein in dysregulating the host antioxidant defense system, it further emphasized the important role of SIRT1/Nrf2 pathway in the host cell for the pathological management of SARS-CoV-2 infection via an antioxidant defense mechanism (Zhang et al., 2022).

Similarly, the Nrf2-dependent antioxidant pathway have been found to be suppressed in the biopsies of COVID-19 patients but interestingly the Nrf2 agonists like dimethyl fumarate (DMF) and 4-ocy-itaconate (4-OI) were reported to induce cellular antiviral effects that could inhibit the replication of SARS-CoV-2 by suppressing the pro-inflammatory response of the SARS-CoV-2 (Olagnier et al., 2020). Nrf2 has also been reported as an important transcriptional repressor of the inflammatory genes in macrophages by blocking the transcription of proinflammatory cytokines, most notably interleukin1β (IL-1β) (Hayes and Dinkova-Kostova, 2014).

The main protease (Mpro) in SARS-CoV-2 responsible for viral polyprotein processing is called 3C-like protease (3CLpro) or 3-chymotrypsin-like-proteases, a highly conserved protease among coronaviruses. It is a cysteine protease that corresponds to Nsp5 of coronavirus and acts as a potential drug target for antiviral therapy against the coronavirus. Several protease covalent inhibitors targeting 3CLpro like CLpro-1, GC376, rupintrivir (formerly AG7088), lufotrelvir (PF-07304814) have already been discovered by structural-based drug designs which are advantageous with low minimum side effects and maximum therapeutic efficacy. Of these, the prodrug PF-07304814 (lufotrelvir) entered clinical trials in September 2020 (Owen et al., 2021). As the Nrf2 pathway plays a significant role in the pathophysiology of both host cells and viruses, Nrf2 modulators have been recommended as promising supplements for the treatment of viral infections by reducing the effects of virus-induced oxidative stress. In 2021, Qi Sun and co-workers discovered oleanolic acid-derived semi-synthetic triterpenoids like bardoxolone and bardoxolone methyl compounds with electrophilic moieties as 3CL pro inhibitors that may covalently bind to the active site cysteine of SARS-CoV-2 3CLpro. These compounds were identified as Nrf2 activators that can inhibit the NF-κB pathway promoting resolution of inflammation, inhibiting viral replication, and thereby facilitating cytoprotection and tissue repair (Kobayashi et al., 2016). Using a murine model of infection and airway epithelial cells, Qu et al. demonstrated that SARS-CoV-2 can alter cellular redox balance and inhibit Nrf2-mediated antioxidant responses. Infection with SARS-CoV2 downregulated Nrf2 protein levels and Nrf2-dependent gene expression, resulting in increased inflammation and disease progression. In addition, mice lacking the Nrf2 gene exhibited worse clinical signs, had increased inflammation, and showed a tendency toward higher lung viral titers, demonstrating that Nrf2 has a protective role during SARS-CoV-2 infection. The results of this study provided a mechanistic explanation for the oxidative unbalance associated with SARS-CoV-2 infection, suggesting that activating Nrf2 by pharmacological agents could be a therapeutic strategy for COVID-19 (Qu et al., 2023). Similar observations have been reported for other viruses. In 2006, Jiang et al. discovered that α-Luminol (monosodium 5-amino-2-3-dihydro-1-4-phthalazine dione), an anti-inflammatory drug extensively used by Russian scientists, was able to suppress oxidative stress induced by the infection of temperature sensitive mutant virus Moloney murine leukemia virus (MoMuLV ts-1) (Jiang et al., 2006). In COVID-19 patients, in addition to viral propagation, the inflammatory response of host cells is also important in determining the disease outcome and fatality. In most viral infections the lethality is found to be associated with the inflammatory response orchestrated by the host immune system through cytokine storm rather than the cytolytic action of the pathogen (Fung et al., 2020; Guan et al., 2020). For the comprehensive management of SARS-CoV-2, it is always advised to introduce an anti-inflammatory and antioxidant therapy to complement an antiviral therapy to control inflammation without altering the host cell’s adaptive immunity against the infected virus.

### Role of PI3K/Akt/Nrf2 pathway in SARS-CoV-2 infection induced inflammation

Cellular homeostasis and responses to stress and inflammation are regulated by Nrf2 through NF-ĸB-dependent pathways. There is compelling evidence that Nrf2 is capable of counteracting NF-ĸB-driven inflammation in many experimental models (Tu et al., 2019). The SARS-CoV-2 infection has similar pathophysiology to SARS-CoV and MERS-CoV infections, with aggressive inflammatory responses strongly implicated in the damage to the lungs. COVID-19 is a multifactorial and complex disease that primarily targets the airway epithelial cells of the respiratory tract, which is characterized by diffused alveolar edema in the lungs, infiltrations of inflammatory cells, epithelial dysfunction, and thrombosis (Song et al., 2020; Bridges et al., 2022).

**FIGURE 2:**
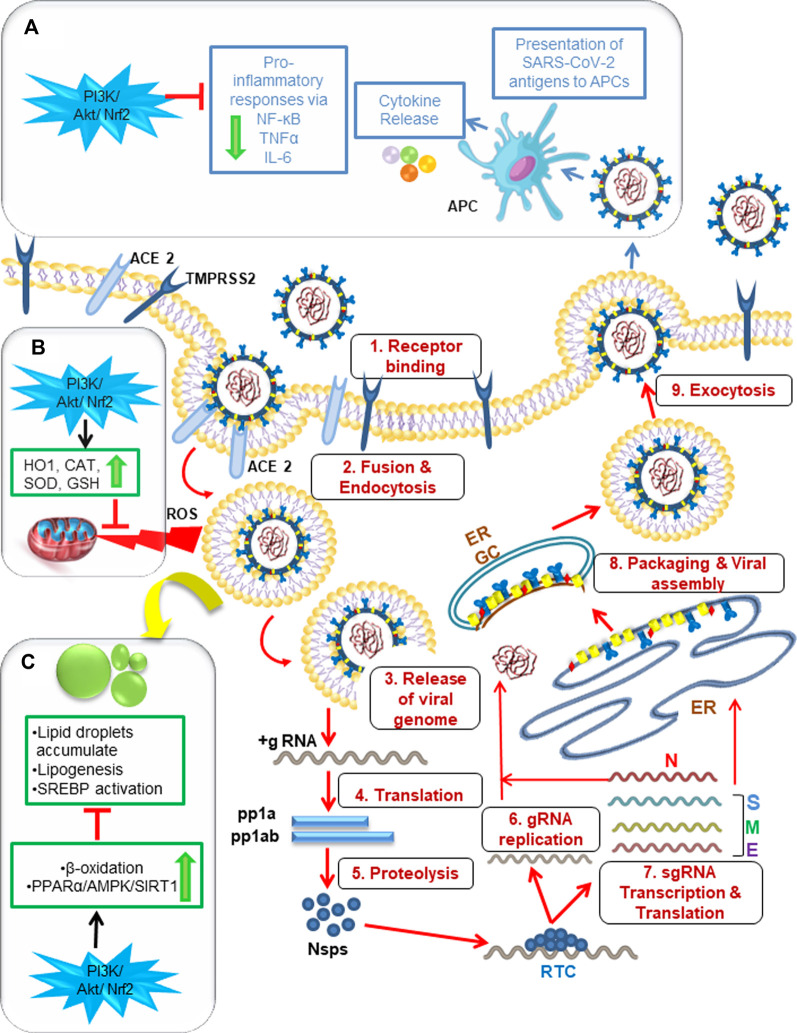
Graphical abstract showing the significance of P13K/Akt/Nrf2 signaling pathway for the management of SARS- CoV- 2 infection via modulating host cell inflammatory responses, antioxidant mechanism and lipid metabolism. 1–9: SARS-CoV-2 enters a target cell by either fusion or endocytosis followed by release of genetic material. Then subsequent events of translation and genome replication occurs leading to the final assembly and egress of virions to infect neighboring cells. **(A)** The released viral particles are recognized by APC of macrophages leading to host inflammatory response via “cytokine storm” that can be downregulated by PI3K/Akt/Nrf2 activators. **(B)** PI3K/Akt/Nrf2 activators can also downregulate ROS-induced oxidative stress produced by SARS-CoV-2 via antioxidant mechanism mediated by the enzymes HO1, SOD, CAT, GSH. **(C)** Lipid droplets can be downregulated via PI3K/Akt/Nrf2 pathway activators by beta oxidation and through the modification of PPARα/AMPK/SIRT1 signaling pathway. ACE2: angiotensin converting enzyme-2, TMPRSS2: Transmembrane serine protease 2, Nsps: non-structural proteins, ERGIC: ER-Golgi intermediate compartment, APC: antigen presenting cells, PI3K: phosphoinositide 3-kinase, Akt: serine/threonine-specific protein kinase B, Nrf2: nuclear factor erythroid 2–related factor 2, PPARα: Peroxisome proliferator-activated receptor, AMPK: AMP-activated protein kinase, SIRT1:Sirtuin, HO1: Heme Oxygenase-1, SOD: Superoxide dismutase, CAT: Catalase, GSH: Glutathione.

Hepatocytes, monocytes, and other endothelial cells are also found to be susceptible to SARS-CoV-2 infection, and evidence suggests that virus-induced hyperinflammation can be triggered by virus-mediated intracellular sensing pathways in cellular targets (Figure 2). Similar to several other viral infections, the alveolar epithelial cells and macrophages recognize pathogen-associated molecular patterns (PAMPs), such as viral RNA, and damage-associated molecular patterns (DAMPs), such as ATP, DNA, and ASC oligomers, using a variety of pattern-recognition receptors (PRRs) during SARS-CoV-2 infection (Huang et al., 2020; Asha et al., 2021). These activated monocytes and polymorphonuclear cells infiltrate into the target cells and cause the release of proinflammatory cytokines and chemokines, like TNF-α, IL-1β, IL-6, CCl2, MCP1 and IP 10 upon interaction of viral particles with antigen-presenting cells (APCs). The release of these indicators of T helper 1 (TH1) cell-polarized response results in severe lung damage and multi-organ dysfunction. IL-1β is known to induce pyroptosis and is also found to be elevated during SARS-CoV-2 infection (Tang et al., 2021). These data strongly suggest that a COVID-19 patient’s disease severity is not only influenced by the virus but also by the host’s immune response.

SARS-CoV-2 infection has been reported to initiate cell death by both apoptosis and necroptosis pathways. In a SARS-CoV-2-infected HFH4-hACE2 (Hepatocyte nuclear factor-3/forkhead homolog 4-human Angiotensin-converting enzyme 2) transgenic mouse model and in the postmortem lung sections of deceased COVID-19 patients, SARS-CoV-2 infection was found to activate caspase-8 to trigger cell apoptosis and inflammatory cytokine processing in the lung epithelial cells. The study further revealed massive inflammatory cell infiltration and pulmonary interstitial fibrosis, typical of immune pathogenesis as the reason of excessive lung damage in those diseased patients (Li et al., 2020). Such findings may assist in the development of specific therapeutic strategies to treat COVID-19.

ACE2-associated lung injury has also been suggested by both SARS-CoV infection and inflammatory cytokines such as IL-1β and TNF-α through enhancement of ACE2 shedding (Haga et al., 2008). In a study on SARS-CoV and human coronavirus NL63 infection, the spike protein was found to modulate ACE2 (Haga et al., 2008; Glowacka et al., 2010). The loss of pulmonary ACE2 function occurs as a result of the loss of catalytically active ACE2 ectodomains.

Ultimately, this resulted in acute lung injury by disrupting the renin-angiotensin system and enhancing inflammation and vascular permeability. The action of disintegrin and metalloprotease 17 (ADAM17, also known as TNF-α cleavage enzyme, TACE) constitutively sheds ACE2 to release enzymatically active soluble ACE2 (sACE2). Since SARS-CoV S protein-induced ACE2 shedding is tightly coupled with TNF-α production in cell culture conditions, it is possible that sACE2 plays a role in the inflammatory response to SARS-CoV and possibly SARS-CoV-2 as well (Haga et al., 2008; Fu et al., 2020). Angiotensin II (Ang II) is a vasoconstrictor that produces oxidative stress via ROS production and elevated blood pressure. In general ACE converts Ang I to Ang II which in turn is converted into Ang by ACE2. Ang remains bound to MAS receptor, a G protein-coupled receptor for Ang, in various tissues including the heart, brain, kidney, etc. To protect against aneurysms by activating PI3K/Akt/Nrf2 pathway (Shimada et al., 2015; Kamel et al., 2018). A recent study identified 34 compounds with anti-SARS-CoV-2 activity that targeted the mTOR/P13K/Akt pathway and DNA-damage response signaling pathways to block SARS-CoV, MERS-CoV and SARS-CoV-2 infection (Garcia et al., 2021). The kinase inhibitor berzosertib also blocked the SARS-CoV-2 at post entry levels in target epithelial cells (Garcia et al., 2021).

Previous studies have demonstrated the crucial role of ACE2/Ang/MAS axis in activating the Akt signaling to manage oxidative stress, inflammation, and hepatic steatosis (Cao et al., 2016) and that Akt inhibitors significantly reduce the ACE2 mediated lipid metabolism, thereby providing insights to manage the SARS-CoV-2 infection-induced metabolic changes in host cells (Cao et al., 2016).

Uncontrolled inflammatory responses known as the cytokine storms have been reported previously in case of SARS-CoV and MERS-CoV infections giving rise to heightened immune response leading to overproduction of proinflammatory cytokines such as the IL-6, TNF-α and IL-1β (Teijaro, 2017). SARS-CoV2 infection is also known to active immune response, more specifically in older adults or those with comorbidity, to a level that can give rise to uncontrolled inflammatory responses (Nile et al., 2020). It is very crucial to control excessive inflammation in COVID-19 patients with severe disease at right time, in absence of which, the condition quickly deteriorates leading to acute respiratory failure, cardiac damage or multi-organ failure (Costela-Ruiz et al., 2020). Naturally occurring phytochemicals, since decades, have been in use as therapeutics to manage diseases with minimal or no side effects. Flavonoids are secondary plant metabolites that have been shown to have anti-viral, anti-inflammatory and immunomodulatory activities (Chen et al., 2018; Hosseinzade et al., 2019; Liskova et al., 2020; Badshah et al., 2021). For example, *Smilax campestris* aqueous extract, containing the catechin and derivatives of quercetin, has been shown to reduce the production of proinflammatory cytokines such as the TNF-α, IL-1β, IL-6, IL-8, and MCP-1 in lipopolysaccharide-activated macrophages derived from THP-1 cells (Salaverry et al., 2020). Hesperetin and chrysin have been shown to have immunomodulatory potential in physiological and pathological conditions through the cellular as well as humoral responses (Sassi et al., 2017). Similarly, many flavonoids have also been demonstrated to exert immunomodulatory activities against human coronaviruses and *in silico* studies have further provided evidence that these flavonoids have potential to bind to ACE2 protein and ultimately inhibit the production of proinflammatory cytokines (Ngwa et al., 2020) thus making them an attractive therapeutic agent. Likewise, rhamnocitrin, a flavonoid extracted from *Nervilia fordii*, has shown its potential to inhibit the endothelial activation (via miR-185/STIM-1/SOCE/NFATc3) which is responsible for excessive cytokine production. Since a similar endothelial activation in case of SARS-CoV or COVID-19 have also been documented, rhamnocitrin may serve as potential modulator of the cytokine storm and effective management of COVID-19 (Lin et al., 2020).

Recent reports suggest the direct or indirect role of the PI3K/Akt signaling pathway in SARS-CoV, MERS-CoV and SARS-CoV-2 infection (Mizutani et al., 2005; Kindrachuk et al., 2015; Sun et al., 2021b). This signaling pathway has also shown to be the target of some flavonoids such as the quercetin, hesperidin, acacetin, geninstein, silibinin and delphinidin among many others (reviewed in (Zughaibi et al., 2021)) wherein they inhibit the deregulated signaling significantly in different types of cancer.

NLRP3 inflammasome has also been shown to be regulated by PI3K/Akt signaling in atherosclerosis and inhibitors of PI3K (GDC0941) and Akt (MK2206) significantly reduced the activation of NLRP3 and expression levels of p-p65/p65. The inhibitors further reduced the mitochondrial ROS in THP-1 cells and mice model (Liu et al., 2021). Since NLRP3 inflammasome is also reported to be activated by SARS-CoV-2, it’s imperative to understand the management of virus induced ROS and inflammatory response by modulating the PI3k/Akt signaling pathways using the anti-inflammatory and immunomodulatory properties of flavonoids. These natural products might be very helpful in minimizing the SARS-CoV-2 complications by regulating inflammatory mediators and endothelial activation by toll-like receptors (TLRs), NLRP3 inflammasome, Nrf2, bromodomain-containing protein 4 (BRD4), or 3CL pro (Liskova et al., 2021).

COVID-19 disease severity has also been correlated with TLR2 and MYD88 expressions, and it has been observed that TLR2 senses the SARS-CoV-2 envelope protein to produce inflammatory cytokines. MyD88, the adaptor protein for TLRs, leads to the activation of NF-ĸB and MAPKs for the production of proinflammatory cytokines. As a result of TLR2 and Myd88 activation, during coronavirus infection, TLR2-dependent signaling leads to the production of proinflammatory cytokines independent of viral entry. In healthy tissues, the TLR-mediated signaling leads to the activation of the PI3K/Akt/Nrf2 pathway for the positive regulation of cell growth (Laird et al., 2009; Zheng et al., 2021). In light of these data, better therapeutic strategies to counter the ongoing COVID-19 pandemic could be developed and effectively manage disease burden.

### SARS-CoV-2 infection and host lipid metabolism

Lipids are one of the fundamental components of a cell that make up the structural building blocks. As a signaling and energy storage molecule, it has a wide range of biological functions. Lipid plays a crucial role in the viral life cycle. The enveloped viruses, like SARS-CoV-2, are surrounded by a lipid bilayer and each step of the viral infection such as fusion of membrane to host cell, endocytosis, viral replication, maturation, and exocytosis utilizes host lipid metabolism (Abu-Farha et al., 2020). The coronaviruses create double-membrane vesicles (DMVs), a membranous structure consisting of viral proteins and some host factor, for viral genome amplification after seizing the intracellular membrane of host cells. Such a lipid micro-environment that contains specific phospholipid composition is ideal for viral replication. Recent studies have shown that an important lipid processing enzyme belonging to the phospholipase A2 superfamily, cytosolic phospholipase A2 enzyme (cPLA2) is crucial for DMV formation and viral replication (Muller et al., 2018).

Fatty acids and cholesterol are the inevitable components of viral replication as they constitute the major component of the viral membrane. Therefore, Acetyl-CoA carboxylase (ACC), fatty acid synthase (FASN), and 3-hydroxy-3-methyl-glutaryl-CoA reductase (HMG-CoA reductase, the major modulators of lipid metabolism, can act as possible antiviral targets against SARS-CoV2 infection (Heaton and Randall, 2011). The recent studies on HIV infection (Kulkarni et al., 2017), hepatitis C virus (HCV) infection (Yang et al., 2008), and Epstein–Barr virus (EBV) lytic and latent infection (Li et al., 2004) confirmed this hypothesis. An increase in the intracellular level of FAS was observed in all these conditions and it was also evident that FAS inhibition impaired the replication of the respiratory syncytial virus (RSV) and other respiratory viruses (Ohol et al., 2015). These findings make this enzyme a novel host-dependent antiviral target. The intracellular levels of fatty acids and cholesterol are regulated by a feedback mechanism mediated by SREBPs (Sterol regulatory element binding proteins), which are bound to the endoplasmic reticulum membrane as inactive precursors. When the cells are deprived of cholesterol, SREBPs are proteolytically cleaved and the active SREBP migrates to the nucleus for the transcriptional regulation of genes responsible for lipid metabolism. This can also be a potential candidate related to lipid metabolism-related antiviral approaches, that can also be modulated by the PI3K/Akt/Nrf2 pathway (Ye and DeBose-Boyd, 2011).

The major cellular receptors of SARS-CoV-2, ACE2 may be expressed in cholesterol-rich domains of lipid bilayer known as lipid rafts that serve as an entry port for certain viruses especially enveloped viruses. An experiment conducted in Vero E6 cells revealed that integrity of lipid rafts was required for productive infection of severe acute respiratory syndrome coronavirus (SARS-CoV) (Lu et al., 2008). The role of peroxisome proliferator-activated receptors (PPARs), belonging to the nuclear receptor superfamily, as an antiviral candidate is a recent matter of investigation during the COVID-19 pandemic. There are mainly 3 subtypes of PPAR receptors: PPARα, PPARγ, and PPARβ/δ that play well-established roles in cellular differentiation, proliferation, energetic homeostasis, glucose, and lipid metabolism. Several *in vitro* and *in vivo* studies revealed that the stimulation of PPAR by natural or synthetic agonists like curcumin, capsaicin, and eicosapentaenoic acid could prevent cytokine overproduction and the inflammatory cascade associated with virus infections (Ciavarella et al., 2020; Fantacuzzi et al., 2022). Pioglitazone, a PPAR agonist, is also proposed as an effective treatment in COVID-19 people affected by type 2 diabetes, cardiovascular complications, and hypertension by reducing inflammatory parameters and also by inhibiting 3CLpro thereby downregulating SARS-CoV-2 RNA synthesis and replication (Carboni et al., 2020). There is evidence that the PPARα/γ-adenosine 5′-monophosphate- (AMP-) activated protein kinase- (AMPK-) sirtuin-1 (SIRT1) pathway and fatty acid metabolism may be involved in influenza A virus (IAV) replication and pneumonia caused by IAV (Bei et al., 2021). These all synergistically work together to inhibit NF-κB signaling and suppress inflammation. Furthermore, Nrf2 and antioxidant response element (ARE) pathways also interact mutually with, PPARα/γ-AMPK to inhibit inflammation, constituting a positive feedback loop (Kauppinen et al., 2013). In adipose tissue, Gamma-oryzanol, the principal bioactive constituent of ice bran, reduced the levels of TNF-α, interleukin-6 (IL-6), and monocyte chemoattractant protein-1 (MCP-1) (Francisqueti-Ferron et al., 2021). Due to its potent and multi-PPAR activity, astaxanthin has been investigated as a therapeutic strategy to regulate inflammatory and immune responses, contrast cytokine storms, and prevent inflammatory effects following COVID-19 (Talukdar et al., 2020).

PI3K/Akt pathway is also known to play a significant role in host lipid biogenesis as revealed by the study on goose hepatocyte where researchers observed that inhibition of the PI3K-Akt-mTOR pathway drastically reduced the lipids accumulation in hepatocytes (Liu et al., 2016) s.

These alternative approaches that are under development, when backed up by clinical trials, can potentially be promising tools for reducing the pathophysiological complications of SARS-COV-2 infections.

## Conclusion and future perspectives

Despite the widespread use of vaccines, the transmission of SARS-CoV-2 infection is on the rise, which enables new variants to emerge frequently. As a consequence of this unprecedented threat in the 21st century, to alleviate case fatality and patients’ symptoms, therapeutic interventions are urgently needed to compliment currently available vaccines. It is still required to conduct studies to develop a universal vaccine against COVID-19, which can neutralize all variants of SARS-CoV-2. Viruses and hosts have a strict interplay that can be destructed through metabolic disruption, which is an attractive novel strategy to combat viral infections. In this regard, broad spectrum antiviral compounds that target PI3K/Akt/Nrf2 signaling pathways to offer host-mediated antiviral responses in every manner, may be considered as the best drug candidates for the future management of COVID-19 and related post-COVID syndromes. Several FDA-approved inhibitors targetting PI3K and Akt are in use in clinical settings (Basile et al., 2022). While most of the inhibitors of PI3K are used to treat some forms of cancer, its utility in viral infections have not been reported adequately. Similarly the Akt inhibitor (Miltefosine) have been in use for Visceral and cutaneous leishmaniasis (Sundar and Olliaro, 2007), however the role in viral infections are still limited to research settings (Sharma et al., 2018). The processes of oxidative stress, inflammation, and changes in lipid metabolism are interconnected and all contribute to both SARS-CoV-2 infection and post-COVID-19 complications. According to the World Health Organization, many patients are experiencing short-to long-term post-COVID-19 sequelae such as cardiovascular, neurological, nephrological, gastro-intestinal, and even psychological effects. A major cause of mortality was reported to be thromboembolism, the cumulative risk product of all the above discussed pathophysiological conditions. PI3K/Akt pathway is known to be an important regulator of coagulation pathways and hence a key player in disease modulation (Shan et al., 2019). Another critical area of concern is the ‘postural orthostatic tachycardia syndrome’ (POTS) occurring after SARS-CoV-2 infection or COVID-19 vaccination. POTS is a condition in which there is an increase in heart rate of at least 30 beats per minute within 10 min of standing. SARS-CoV-2 infected people and those who have been vaccinated against COVID-19 have had an increased risk of cardiovascular diseases (CVDs), but it is unclear if this is due to the virus infection or the vaccination (Blitshteyn and Fedorowski, 2022). The situation is unfortunate as there is no complete cure. However, the overall immunity can be strengthened in order to compete with viral infections. Reports indicate that the best natural immunity boosters are functional foods that offer health benefits beyond their nutritional values. Incorporating functional food ingredients in diet can activate cell survival pathways like PI3K/Akt/Nrf2, that can reduce long-term health risks associated with COVID-19.

Overall, in this review article, we propose potential anti-inflammatory and antioxidant therapies that can also regulate lipid metabolism by targeting transcription factor Nrf2 via the PI3K/Akt signaling pathway. Combined, the research discussed in this article strongly suggests that activating Nrf2 could be a promising strategy for combating COVID-19. Further investigations along this line are needed to develop efficient counteracting strategies to ameliorate disease severity and improve treatment outcomes, especially for patients with underlying complications.
